# A partnership approach to providing on-site HIV services for probationers and parolees: a pilot study from Alabama, USA

**DOI:** 10.7448/IAS.19.4.20868

**Published:** 2016-07-18

**Authors:** Bronwen Lichtenstein, Brad Wayne Barber, The West Alabama AIDS Outreach Partnership Group

**Affiliations:** 1 Department of Criminal Justice, The University of Alabama, Tuscaloosa, AL, USA; 2 Tuscaloosa Probation and Parole Office, Tuscaloosa, AL, USA School of Social Work, The University of Alabama, Tuscaloosa, AL, USA; 3 West Alabama AIDS Outreach, Tuscaloosa, AL, USA

**Keywords:** community-based HIV partnership, probation and parole, HIV education and testing

## Abstract

**Introduction:**

HIV in the United States is concentrated in the South, an impoverished region with marked health disparities and high rates of incarceration, particularly among African Americans. In the Deep South state of Alabama, a policy directive to reduce prison overcrowding has diverted large numbers of convicted felons to community supervision. Probation and parole offices have yet to provide the HIV education and testing services that are offered in state prisons. This study sought to implement on-site HIV services for probationers and parolees through an intersectoral programme involving law enforcement, university and HIV agency employees. The three main objectives were to (1) involve probation/parole officers in planning, execution and assessment of the programme, (2) provide HIV education to the officers and (3) offer voluntary pretest HIV counselling and testing to probationers and parolees.

**Methods:**

The partnered programme was conducted between October and December 2015. Offenders who were recently sentenced to probation (“new offenders”), received HIV education during orientation. Offenders already under supervision prior to the programme (“current offenders”) learned about the on-site services during scheduled office visits. Outcomes were measured through officer assessments, informal feedback and uptake of HIV services among offenders.

**Results:**

A total of 86 new and 249 current offenders reported during the programme (*N*=335). Almost one-third (31.4%) of new offenders sought HIV testing, while only 3.2% of current offenders were screened for HIV. Refusals among current offenders invoked monogamy, time pressures, being tested in prison, fear of positive test results and concerns about being labelled as gay or unfaithful to women partners. Officers rated the programme as worthwhile and feasible to implement at other offices.

**Conclusions:**

The partnership approach ensured support from law enforcement and intersectoral cooperation throughout the programme. HIV training for officers reduced discomfort over HIV and fostered their willingness to be active agents for referral to HIV services. Voluntary testing was enhanced by the HIV employee's educational role, particularly during orientation sessions for new offenders. The almost one-third success rate in HIV testing among new offenders suggests that future efforts should concentrate on this group in order to maximize participation at the probation and parole office.

## Introduction

The United States has the highest rate of incarceration in the world, a distinction that disproportionately affects men of colour who are at elevated risk of HIV/AIDS [1]. In Alabama, the site of this pilot study, state prisons are populated at 197% of capacity and are reputed to be the most crowded and unsafe facilities in the nation [2]. In the wake of reforms to reduce prison overcrowding, non-violent offenders are being diverted to community supervision in order to serve suspended sentences [3]. Compared to state prisons, which offer HIV prevention education and testing for inmates, community correctional offices lack such programmes and are ill-equipped to provide HIV services in the face of increasing demand. An underserved population therefore lacks access to HIV services even though public health authorities have urged “people who work in communities to play an active role in implementing the [national prevention strategy] to reduce HIV rates in high-risk groups” [4]. The two trends – prison diversion programmes for convicted offenders and federal goals to reduce HIV rates in the South – have created high demand for on-site HIV services at probation and parole offices in Alabama as an underserved state.

This article describes an intersectoral partnership between the first author (a medical sociologist), West Alabama AIDS Outreach (WAAO) (an agency that provides social support services to clients and HIV prevention to the community), and the second author, who is a probation officer at the Tuscaloosa County Probation and Parole Office. The goal of the study was to provide HIV education for officers and HIV services for offenders under community supervision. The partnership built on emerging evidence from three studies that support probation and parole offices as key sites for HIV intervention. In the most pertinent study [5], probationers were significantly more receptive to receiving on-site HIV services than being referred to a community health centre for testing. In two behavioural interventions, probationers engaged in less drug use and risky sex after participating in an on-site programme and, among women probationers, evidenced higher rates of protected sex [6,7]. El Bassel *et al*. [7] concluded that probation and parole offices are “ideal venues for engaging low-income women in order to achieve a high public health impact.” On-site HIV services are valuable for another reason: convicted offenders are at greater risk of HIV in the free world than if they are behind bars [8]. However, probation and parole offices are rarely used for HIV programmes [6,9], a deficit that the authors hoped to correct for probationers and parolees at the county scale. The partnership therefore sought to identify whether or not on-site HIV services could be implemented successfully as measured by officer support and uptake among offenders for pretest counselling and HIV testing.

## Methods

### Setting and demographics

The programme was conducted from October to December 2015 at the Tuscaloosa County Probation and Parole Office, one of 61 state offices in Alabama. The plan involved enlisting officers to facilitate the programme, an HIV educator/tester to provide on-site services and offenders to volunteer for pretest counselling and testing over a three-month period. Of eight probation officers at the office during the study (a full complement), all were aged between 26 and 61 years and 62% were both White and male. These officers participated in the programme in equal measure. Only half of this group (50%) had more than five years’ experience, and none had received HIV-related training during their careers. With an average caseload of 184 offenders, the officers had a higher workload than counterparts in other states [10] but were willing to be educated about HIV and to facilitate access to the programme for probationers and parolees at the office.

The offenders who participated in the programme were drawn from a full cohort of 1,674 probationers and parolees under supervision at the office in 2015. This cohort was mostly male (85%), aged between 25 and 35 years old (66%) and predominantly African American (58%). With a 2:1 ratio of Whites and African Americans in the county [11], these demographics reflect the over-representation of young men of colour in US corrections [12]. Most convictions involved drug possession (44%), property crimes (26.8%), violent crimes (19.2%) and sex offenses (6.2%). The preponderance of drug-related crime indicates the level of HIV risk that the programme sought to address. As required by law, all offenders were required to undergo random searches, home visits and drug testing on a regular basis. Alabama law does not require HIV disclosure to officers and this information was neither available nor sought by the authors.

### Design and methods

#### Programme development

The second author's diverse roles as a doctoral candidate in social work, senior probation officer and former police officer and correctional officer played a key part in gaining support from law enforcement and state administrators, and in conceptualizing and managing the logistics of the pilot study. Prior to the study, this author became aware of the surge in non-violent offenders being diverted to community supervision without ready access to HIV services. He jointly embarked on the present venture with the first author, who drew upon her contacts with WAAO for the project. In the formative stages, the second author collected informal feedback from co-workers and offenders in order to develop suitable methods for the programme and to ensure the study did not interfere with daily operations at the probation office. He was on duty and supervised the project while the HIV services were on site.

#### Design

The Alabama Board of Pardons and Paroles and The University of Alabama approved all aspects of the programme. The officers signed a consent form that included a written explanation of the purpose of the study and instructions for two pen-and-paper surveys titled *Officers’ knowledge and attitudes toward HIV* and *Assessment of full programme*. The offenders were neither interviewed nor surveyed because of the potential risks of divulging arrest-worthy information in a supervised setting. The programme consisted of two educational HIV sessions for officers, four half-days of voluntary access to on-site HIV services for current offenders during scheduled reporting periods, and four educational HIV sessions and access to on-site HIV services for new offenders who attended orientation days in order to complete forms for supervision. WAAO provided on-site education, pretest counselling and testing services for up to three hours per time, for a total of 22 hours. Outcomes were determined by the feasibility of the programme, the level of officer support and the utilization of HIV services.

The analytical plan consisted of comparing levels of uptake for pretest counselling and HIV testing for each group of offenders. WAAO provided demographic details for both new and current offenders who used the service but was not authorized to supply the results of HIV tests to non-WAAO personnel. For the survey analysis, the authors calculated the number and percent of correct responses on the officers’ knowledge and attitudes survey and scored the ratings for scaled items in the programme assessment. These scores were matched with narrative comments from each survey for illustrative purposes.

#### Officers

In segue to the full programme, the first author provided officers with updated information on HIV transmission, rapid testing, viral loads, antiretroviral treatment (ART), pre- (PrEP) and post-exposure prophylaxis (PEP), and occupational safety. These sessions included brainstorming to fine-tune the programme and a 15-item scaled/textbox survey to gauge what officers had learned about HIV transmission, prevention and treatment modalities. At the end of the full programme, the officers provided feedback in a 23-item scaled/textbox survey on HIV knowledge, safety concerns, programme effectiveness, time demands and future directions. The officers also provided informal feedback on offenders’ interest in the programme.

#### Offenders

Probationers and parolees were organized into two groups according to normal reporting procedures for the office (new offenders attended fortnightly orientation sessions; current offenders reported for mandated monthly visits). Within this framework, new offenders met with the HIV educator in groups of 16 to 22 people, where HIV risks, prevention, testing and treatment were discussed in roundtable fashion. Although these discussions were an integral part of orientation, the officers were not present, and volunteers were taken to a private office at the rear of the building for testing. In contrast, current offenders received a flyer with sign-in forms and an invitation to speak to their assigned officer about the HIV services. The flyer included a coupon for a bag of free condoms. If not initiated by the offender, their officer raised the topic during the interview and accompanied volunteers to the WAAO office for pretest counselling and/or testing. The educator kept a record of all visits by group assignment and provided aggregate information on offenders’ demographics and points of contact. All eight officers knew at least one offender on their caseload who had self-disclosed as HIV-positive. However, privacy protections for the study meant that actual test results could not be made available for analysis.

## Results

### Officer knowledge

Prior to the updates, most officers (75%) were aware that HIV could not be transmitted through casual contact with people or objects (e.g. shaking hands or hugging someone with HIV, drinking fountains, toilet seats, or surfaces). Only half (50%) of the group knew that spitting and vomit, sometimes encountered in the field, did not pose a threat, and that unprotected sex accounted for 97% of HIV infections in Alabama. Few officers (25%) were aware that ART, if taken as prescribed, greatly reduces the likelihood of infecting sexual partners. Officers did not realize that a daily dose of ART could prevent HIV infection or that a 28-day course of ART prevented transmission after occupational exposure. In short, much of the information about ART, PrEP and PEP was completely new to the officers, who reported that they would feel more comfortable around offenders in light of this information (Table 1).

**Table 1:** Officers’ knowledge and attitudes towards HIV (*N*=8)

	Correct	
	
Item	No.	%
1. Before today, I was aware that:		
*i*. *HIV cannot be acquired from:*		
Surfaces such as tables, door knobs and bench tops	7	(88)
Air, water or swimming pools	7	(88)
Touching, hugging or shaking hands	6	(75)
Toilet seats, drinking fountains, surfaces or food sources	6	(75)
Coughing or sneezing	4	(50)
Spitting or vomiting	4	(50)
*ii*. *Antiretroviral therapy (ART) prevents HIV by:*		
Reducing viral loads (infectiousness) in patients	2	(25)
Offering protection prior to exposure, if taken daily (PrEP)	0	(0)
Offering protection after exposure, if taken daily for 28 days (PEP)	0	(0)
2. After today, I feel more confident about:		
Protecting myself in the event of occupational exposure	8	(100)
The benefits of ART for HIV treatment and prevention	8	(100)
Interacting with HIV-infected offenders	7	(88)^a^

a HIV status was known if offenders self-disclosed during interviews.

### Offender uptake

#### New offenders

A total of 86 offenders participated in group sessions during orientation. The educator opened each session by asking why probationers and parolees are at greater risk of HIV than the general public [5,13]. Modes of HIV transmission and prevention strategies were then discussed with the group. Not only were these sessions were highly interactive, but several offenders felt comfortable enough to discuss personal risk factors such as drug use and being diagnosed with sexually transmitted infections. After learning that ART is life-saving and testing does not involve blood draws, 32 offenders volunteered for pretest counselling. A total of 27 of these offenders (52% African American, 70% male) were tested and offered a bag of free condoms and a certificate of recognition. This total represented 31.4% of the 86 new offenders in the study. Informal feedback was positive: “You should do this all the time,” and “It's an eye-opener that you can get free testing and it's not a blood draw.”

#### Current offenders

A total of 249 offenders reported to their officers while the HIV educator was on site. While few offenders produced the flyer as invited, 29 opted to visit the educator once the topic was raised by their officer. All 29 volunteers received pretest counselling during this visit. A total of eight offenders (100% male, 75% African American) were then tested, after which they were offered a certificate and bag of condoms. This total represented 3.2% of 249 current offenders. Some offenders refused testing and/or the condoms for fear that wives and girlfriends would discover that they had been unfaithful or were having sex with men. As reported by officers, commonly-stated reasons for refusal also related to monogamy, time pressures, “being too embarrassed,” “being killed by my girlfriend,” “afraid of bad results,” “already been tested” [in prison or during pregnancy] and “wanting to get out of the probation office.” The claims of being tested in prison could not be verified because offenders’ HIV status was neither sought nor disclosed within the context of the study.

**Figure 1:**
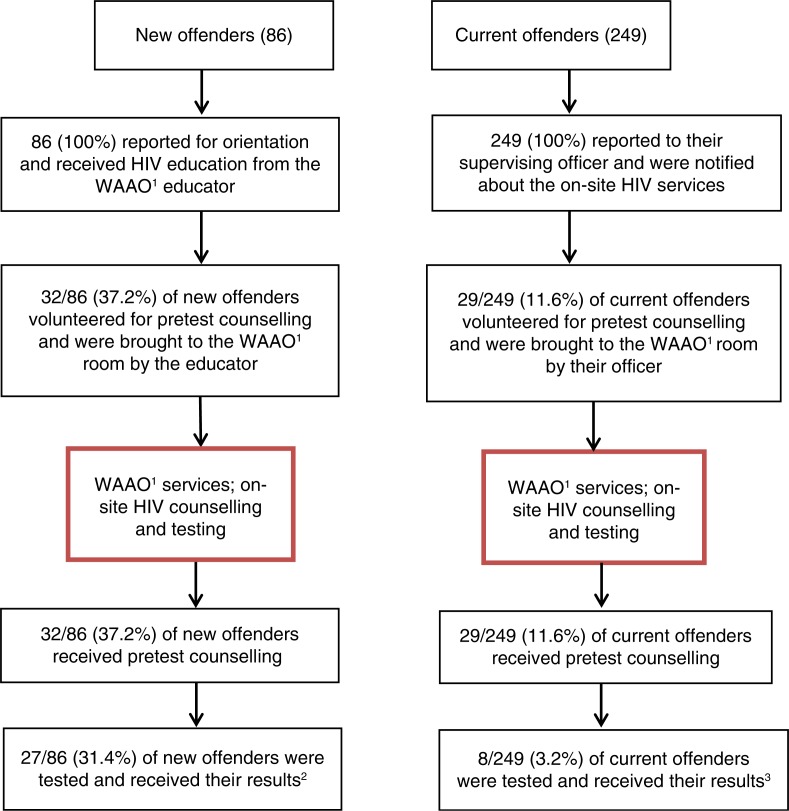
Flow chart for new and current offenders offered HIV education, pretest counselling and testing at the Tuscaloosa County Probation and Parole Office (*N*=335). ^1^West Alabama AIDS Outreach. ^2^New offenders tested: 14 African Americans (12 men; 2 women); 13 Whites (7 men; 6 women). ^3^Current offenders tested: 6 African American men; 2 White men. No women were tested.

Figure 1 presents a flow chart of the overall programme design and points of contact between officers, HIV educator and the offenders who received education, pretest HIV counselling and testing. The chart also provides a side-by-side comparison of the results for each group and stage of the programme. Most testers were male and African American, a result that is consistent with the gender and racial demographics for all offenders under supervision at the Tuscaloosa County Probation and Parole Office.

### Post-programme assessments

Officers rated the programme as successful, wanted it to continue and sought expansion to other sites (Table 2). Narrative comments showed strong support for including both officers and offenders in the programme: “It benefits everyone”; “Keep coming to our office every month during reporting”; and “All law enforcement should be involved.” The level of effort was deemed reasonable, although one officer referred to “one more thing” in terms of work overload. With regard to knowledge, the officers felt more informed about HIV transmission, prevention strategies and treatment options, and related the benefits of such knowledge to having fewer concerns over occupational safety (e.g. “new information about available treatment lessened my fear of transmission”; “knowing that there is a pill available is reassuring”; and, “having back-up medicine means HIV won't get into my system after cleaning up spilled blood”). Such concerns were still evident in response to items about physical confrontations that involved biting, spitting and pat-down searches.

**Table 2:** Officers’ assessments of the full programme (*N*=8)

	Agree	
	
Item	No.	%
1. *As a result of the programme, I have become more:*		
Knowledgeable about HIV risks, prevention and treatment	8	(100)
Confident about sharing this knowledge with offenders	8	(100)
Satisfied with the quality of HIV services	8	(100)
Likely to refer offenders for HIV education and testing	8	(100)
Likely to share HIV knowledge with colleagues	2	(25)
2. *In terms of personal safety, I am:*		
Less fearful about becoming HIV-infected during searches and arrests	7	(88)
Less concerned about supervising HIV-infected offenders	5	(63)
3. *Type of contact that still concerns me:*		
Biting	4	(50)
Pat-down searches	4	(50)
Spitting	3	(38)
Shaking hands	1	(9)
Handcuffing	1	(9)
4. *Future directions:*		
The programme should become permanent	8	(100)
The programme should be offered statewide	8	(100)
Officers should be educated about HIV on a regular basis	6	(75)^a^
HIV education should be mandatory for drug users and new offenders	5	(63)^a^
The programme should be publicized widely	4	(50)^a^

a Not all officers completed these items; percentages reflect the level of response for each item.

## Discussion

Community supervision in the United States has expanded through early release and diversion programmes that are designed to reduce mass incarceration on a nationwide basis [3,14]. In response to this transition at the state level, Tuscaloosa's intersectoral partnership provided on-site HIV education, pretest counselling and rapid testing for probationers and parolees in the county. The programme was executed with existing personnel, infrastructure and resources with a view to implementation at other locations. With officers as recipients of HIV education, but also as agents of change, the plan is a beginning point for reducing HIV stigma, raising awareness and alleviating fears of occupational exposure that are associated with harsher treatment of HIV-infected arrestees [15,16].

Tuscaloosa's intervention can help guide similar efforts for HIV services among community-supervised offenders in the United States and could also be adopted in other countries that have mounted efforts to reduce prison crowding through community-based alternatives [17]. Raynor's [18] review of the probation system in the United Kingdom has endorsed officers’ importance in providing community-supervised offenders with access to social services, drug treatment and other programmes. In the United States, the trend towards partnerships between law enforcement and public health is seen in a growing number of police departments that allow officers to carry Naloxone to prevent or reverse drug overdose [19]. A point of relevance for populations being served by such programmes is that offenders are disproportionately poor, minority-ethnic and socially disadvantaged in many regions of the world, including the Global South [20]. The take-home message is that law-enforcement/public health collaborations can be immensely helpful for bringing local services to these underserved populations and for fostering intersectoral goodwill.

Capacity-building is a core principle of partnership work that seeks to respond to local needs, build trust between agencies and be sustainable over time [21]. The Tuscaloosa programme was developed through trial and error in the absence of formal models to guide such efforts on behalf of probationers and parolees in Alabama. It is noteworthy that Gordon *et al*.'s [5] randomized controlled trial of HIV testing at on-site/off-site locations was also prompted by the absence of US-based models for community-supervised offenders [6]. This trial established the value of on-site services for HIV testing for these offenders, but did not provide a smaller, community-based model to guide a local effort. Even with cash incentives, the rate of refusal during the trial was quite high (45%), with offenders citing familiar reasons such as monogamy, prior testing, incarceration and antipathy towards testing.

In Tuscaloosa's case, feedback loops, inter-agency liaison and institutional support ensured that the programme was feasible and did not fail when offenders’ interest was low and adjustments to the original design, such as group sessions for new offenders, were called for. These efforts produced a results-based “best practices” model to guide future efforts and new procedures at the office, such as regular HIV updates for existing employees and informational packets and HIV training for new employees. The programme did not offer financial incentives to offenders, which might have improved the level of uptake for testing. A modest level of financial support will be required if the programme is to be truly sustainable (e.g. for incentives and supplies) and for broader adoption by probation and parole offices at other locations.

The programme indicated the importance of pilot studies in guiding novel efforts, and for reflecting on environmental and other barriers to HIV testing. On this point, 82% of current offenders declined to receive pretest HIV counselling and testing on site. This rate of refusal is higher than for Gordon *et al*.'s trial [5] in which probationers and parolees received $20 for baseline HIV assessments. In the absence of monetary incentives, the presence of uniformed officers with side-arms and powers of arrest might have deterred anxious or wary offenders who were invited to participate in the programme. Criminal justice settings are inherently coercive [22], notwithstanding assurances by study personnel that participation is entirely voluntary. Other factors, such as fear of a positive result, mistrust of health workers, HIV stigma and general lack of awareness about risk factors, could play a role as well. Despite support for HIV screening in prior research with probationers and parolees [5,13], these local concerns suggest that stigma-related avoidance will prevail without strategies to improve uptake among convicted offenders. On a related point, WAAO's health fairs at local churches and workplaces, and for residents of public housing, rarely lead to voluntary uptake, indicating the difficulty in providing testing services through community outreach.

The results of the new offender component of the programme were more encouraging, with almost one-third of this group being tested (31.4%). As noted in Figure 1, the participants were predominantly male and African American. Such characteristics are consistent with the demographic profile of all supervised offenders at the office and more broadly for the at-risk population for HIV in the southern United States [1,4]. WAAO's expertise was instrumental in educating new offenders about the benefits of oral testing and ART during orientation. This expertise also made a positive difference among 29 current offenders who received pretest counselling, with eight men from this group being tested for HIV. As education was the key to participation, WAAO-led sessions about risk factors, safer sex and ART will be provided for all probationers and parolees who attend orientation. This step will ensure that every offender has access to on-site HIV services when they first report for supervision. Familiarization with the on-site services could also improve uptake during regular reporting periods, especially if officers issue a reminder that the services are available.

It seems obvious that cash incentives could enhance uptake for HIV testing as a major goal of the programme. In prior research, two cash-incentivized interventions for women participants in an HIV risk reduction programme [7] and for probationers and parolees in two US states [5] yielded participation rates of 62 and 55%, respectively. Closer to home, the value of cash incentives was evident during a Tuberculosis outbreak in a neighbouring Alabama county, where testing was unsuccessful until health officials offered $40 per person for screening and a follow-up visit to collect results [23]. Probation/parole officers in the Tuscaloosa programme were certain that gift cards or cash would improve HIV testing rates because: “Offenders are poor – why get tested for nothing when you can get $40 to 60 for donating plasma down the road?” Nevertheless, while the challenge remains in terms of maximizing uptake on a sustained basis (interventions that offer monetary incentives tend to be short-lived), the pilot programme has raised awareness about HIV risk, the availability of on-site services and the benefits of outreach to vulnerable offenders who could otherwise slip through the cracks without these services at their disposal.

### Limitations

The shortcomings of this pilot study include the focus on a probation and parole office at a single location. Adding a second group to the programme, and conducting statistical analyses for comparison, would improve the scientific value of the study. In terms of efficacy, the authors underestimated the level of resistance to HIV testing among convicted offenders who were often misinformed about HIV (e.g. “you get AIDS from cats”), and opposed to the idea of blood draws. For this reason, the educational and pretest counselling component was often an endpoint to raising awareness about risk factors, methods of prevention, the benefits of rapid testing (to replace blood draws) and being tested in the future. Blood draws are especially unpopular among African Americans whose aversion to needles is attributed to the Tuskegee Syphilis Study and its antecedents [24]. Direct input from offenders (e.g. in surveys) was also lacking because of bureaucratic concerns over legal liability. Community-supervised adults are a protected population under the Federal Office of Human Research Protection and ethics approvals covered only officer surveys, basic demographics and points of contact for offenders who received education, pretest counselling and testing. A final limitation relates to the presence of a single WAAO employee who was responsible for pretest counselling, rapid testing and returning test results before volunteers left the office. Some volunteers could not be accommodated because of time constraints (each visit took at least 20 minutes per person); having a second WAAO employee thus could increase the rate of testing for the programme. Further resources are clearly needed in order to increase the capacity of the programme and for scale-up in the future.

## Conclusions

The national trend of reducing prison overcrowding by sentencing convicted felons to community supervision calls for intersectoral HIV outreach to probationers and parolees. The practical challenge of bringing voluntary HIV services to this population helps to explain the dearth of on-site US-based programmes to date. Even with motivated teamwork, conducting evidence-based HIV programmes in high HIV stigma contexts within the budgetary constraints of state government and public health spending calls for considerable ingenuity for the criminal justice system's most under-resourced agency [25]. The current programme provides evidence that public-health-criminal justice partnerships are both feasible and timely for implementing HIV prevention programmes at accessible locations and could help to transform the HIV landscape of at-risk communities with high rates of criminal justice involvement.

## Authors' information

Dr. Bronwen Lichtenstein is a Professor of Sociology at The University of Alabama. She has studied HIV/AIDS for the past 23 years in New Zealand and the United States, and collaborated with Mr. Barber in HIV-related research involving probationers and parolees that informed the current programme. Mr. Brad Barber is a doctoral candidate in the School of Social Work at The University of Alabama, with a specialty in HIV/AIDS and law enforcement. He was a police officer in Tuscaloosa County from 2007 to 2009, a correctional officer for the Alabama Department of Corrections from 2010 to 2011 and is currently employed at the Tuscaloosa County Probation and Parole Office. The West Alabama AIDS Outreach Partnership Group provides comprehensive support services and HIV outreach throughout West Alabama, USA.
